# Col. Charles Smart, M.D.

**Published:** 1905-05

**Authors:** 


COL. CHARLES SMART, M.D., senior assistant surgeon-general United States army, died April 23, 1905, at Saint Augustine, Fla., aged 64 years. Dr. Smart served as assistant surgeon 63d regiment of infantry N. Y. volunteers, in which he was commissioned October 27, 1862. He was promoted to assistant surgeon United States army March 30, 1864, and assigned to duty as medical inspector of the second army corps. He was present with the Army of the Potomac during all of its important campaigns, from the autumn of 1862 until the end of the civil war. He continued in the regular medical service of the army from his entrance into the same until his death, serving in several Indian campaigns, in the Spanish war and later in the Philippines. He was the fourth in rank of the colonels of the army of all corps and regiments at the time of his death, and was one of the best known and greatest respected officers of the medical corps. It is more than likely that Col. Smart would have become surgeon-general some time ago except for his impaired hearing, an infirmity caused by exposure in the line of duty.

